# Study on a Traditional Chinese Medicine constitution recognition model using tongue image characteristics and deep learning: a prospective dual-center investigation

**DOI:** 10.1186/s13020-025-01126-w

**Published:** 2025-06-12

**Authors:** Yongyue Liu, Linmiao Fan, Mei Zhao, Dongshen Wei, Menglan Zhao, Yihang Dong, Xiaoqing Zhang

**Affiliations:** 1 School of Life Sciences, Beijing University of Chinese Medicine, No. 11, North 3rd Ring Road East, Beijing, 100029 China; 2 Tsinghua University Institute of Advanced Equipment (Tianjin), Tianjin, 300300 China

**Keywords:** TCM constitution, Tongue diagnosis, Deep learning, Neural network

## Abstract

**Purpose:**

The objective of this study was to develop a quantitatively analyzed Traditional Chinese Medicine (TCM) constitution recognition model utilizing tongue fusion features and deep learning techniques.

**Methods:**

A prospective investigation was conducted on participants undergoing TCM constitution assessment at two medical centers. Tongue images and corresponding TCM constitution data were collected from 1374 participants using specialized equipment. Both traditional and deep features were extracted from these images. Significant features associated with constitutional characteristics were identified through LASSO regression and Random Forest (RF). Eight machine learning algorithms were employed to construct and evaluate the efficacy of the models. The highest-performing model was selected as the foundational classifier for developing an integrated tongue image feature model. Model performance was comprehensively evaluated using accuracy, precision, recall, F1 score, and area under the curve (AUC).

**Results:**

Analysis revealed 11 critical traditional tongue image features and 26 deep tongue image features. Three datasets were constructed: traditional tongue image features, deep tongue image features, and a fusion feature dataset incorporating both. The multilayer perceptron (MLP) model combining traditional and deep features demonstrated superior performance in TCM constitution classification compared to single-feature models. In the training phase, the model achieved an accuracy (ACC) of 0.893 and an AUC of 0.948. On the test set, it achieved an ACC of 0.837 and an AUC of 0.898, with sensitivity and specificity of 0.680 and 0.930, respectively, indicating excellent generalization ability.

**Conclusions:**

This study successfully developed an intelligent TCM constitution recognition model that overcomes the limitations of traditional methods and validates the value of tongue images for accurate constitution recognition.

## Introduction

Constitution is one of the unique concepts in Chinese medicine and is a unique physiological characteristic of the human body that is formed under the influence of a variety of factors including genetics, environment and lifestyle [1]. According to the doctrine of constitution in Chinese medicine, constitution is an important factor influencing the occurrence of diseases, and the difference in constitution of an individual determines his or her propensity for diseases when facing external pathogenic factors [2]. There are two main traditional methods of body identification. One is a comprehensive assessment based on the four TCM diagnostic methods of inspection, smelling, inquiring, and palpation, which involves observing the subject’s face and tongue, asking about medical history, current symptoms, and daily habits, and determining the type of constitution by palpation of the pulse [3]. The second is to determine the constitution by asking the subject to fill in an assessment scale. In 2006, based on the theory of TCM constitution, Professor Wang Qi and his research team developed the Constitution in Chinese Medicine Questionnaire (CCMQ), which was incorporated by the Chinese Society of Traditional Chinese Medicine (CSTCM) into the standards of Classification and Determination of Physical Qualities in Chinese Medicine (CCMQ) in 2009 as a tool for the classification and determination of physical qualities [4]. After years of clinical use, CCMQ has been widely recognised by the TCM community. Convenient and accurate identification of body mass is crucial for enhancing the clinical efficacy of Chinese medicine and the development of personalised treatment plans. It not only helps doctors to understand the overall health condition of the subjects more deeply, but also has positive significance in preventing diseases and maintaining health [5]. However, traditional methods of constitution identification are highly dependent on the subjective judgement of doctors and are easily influenced by personal awareness and clinical experience, resulting in a lack of consistency in the determination of results. In addition, the questionnaire has many questions, is time-consuming to answer, and complicated to calculate the scores assigned to the entries, thus making the determination of body type less precise [6].

In view of the above challenges, it is particularly important to explore more objective and quantitative tools for identification. Tongue diagnosis occupies an important position in Chinese medicine diagnostics, and is both a key basis for clinical dialectical analysis and an important reference for constitution identification [7]. Individual constitution characteristics can be revealed through different states of tongue colour, tongue shape, tongue coating colour and tongue coating texture, and these characteristics play a crucial role in TCM constitution identification [8]. Modern research has confirmed the close connection between constitution and tongue image, and that changes in body functions due to differences in constitution can be captured in time through the tongue image [9]. For example, subjects with Yang-deficiency quality often show characteristics such as pale tongue colour and large tongue size; while wetness-heat type may be accompanied by yellowish and greasy tongue coating.

In recent years, with the rapid development of big data and artificial intelligence technology, deep learning technology has been widely used in tasks such as medical image segmentation, disease diagnosis, and medical image processing, and has significantly improved the accuracy of disease diagnosis in the clinic by virtue of its excellent feature extraction and classification capabilities [10–12]. The deep learning algorithm analyses the tongue images to achieve automated recognition and classification of tongue texture and tongue coating features, providing a more accurate and quantitative diagnostic tool [13]. Meanwhile, models constructed using a variety of machine learning algorithms demonstrated high accuracy in TCM constitution recognition, foreshadowing the potential development of more efficient end-to-end deep learning models in the future [14]. Li et al. proposed a deep learning model based on tongue diagnosis for the diagnosis of gastric cancer (GC) [13]. This study collected tongue images from both gastric cancer patients and non-cancer participants, employing 16S rDNA sequencing to analyse the microbial community present in the tongue coating. The establishment of this artificial intelligence deep learning model revealed that the tongue diagnosis-based model achieved an area under the curve (AUC) value ranging from 0.88 to 0.92 during internal validation, and an AUC value between 0.83 and 0.88 in independent external validation, significantly outperforming traditional blood biomarkers. In a related study, Lu et al. found that for the screening of liver fibrosis [15], the tongue image-based deep learning model recorded accuracies of 0.845 (95% CI 0.79–0.90) in the validation set and 0.814 (95% CI 0.76–0.87) in the test set, with the negative predictive value (NPV) exceeding 90% in both groups. This model demonstrated superior performance compared to the FIB-4 score across all parameters, achieving an NPV of 94.4% when used in conjunction with the FIB-4. From this point of view, deep learning techniques have a broad application prospect in tongue diagnosis and body mass identification, but further expansion of the dataset size and optimisation of the feature extraction method are still needed to improve the generalisation ability and practicality of the model.

**Fig. 1:**
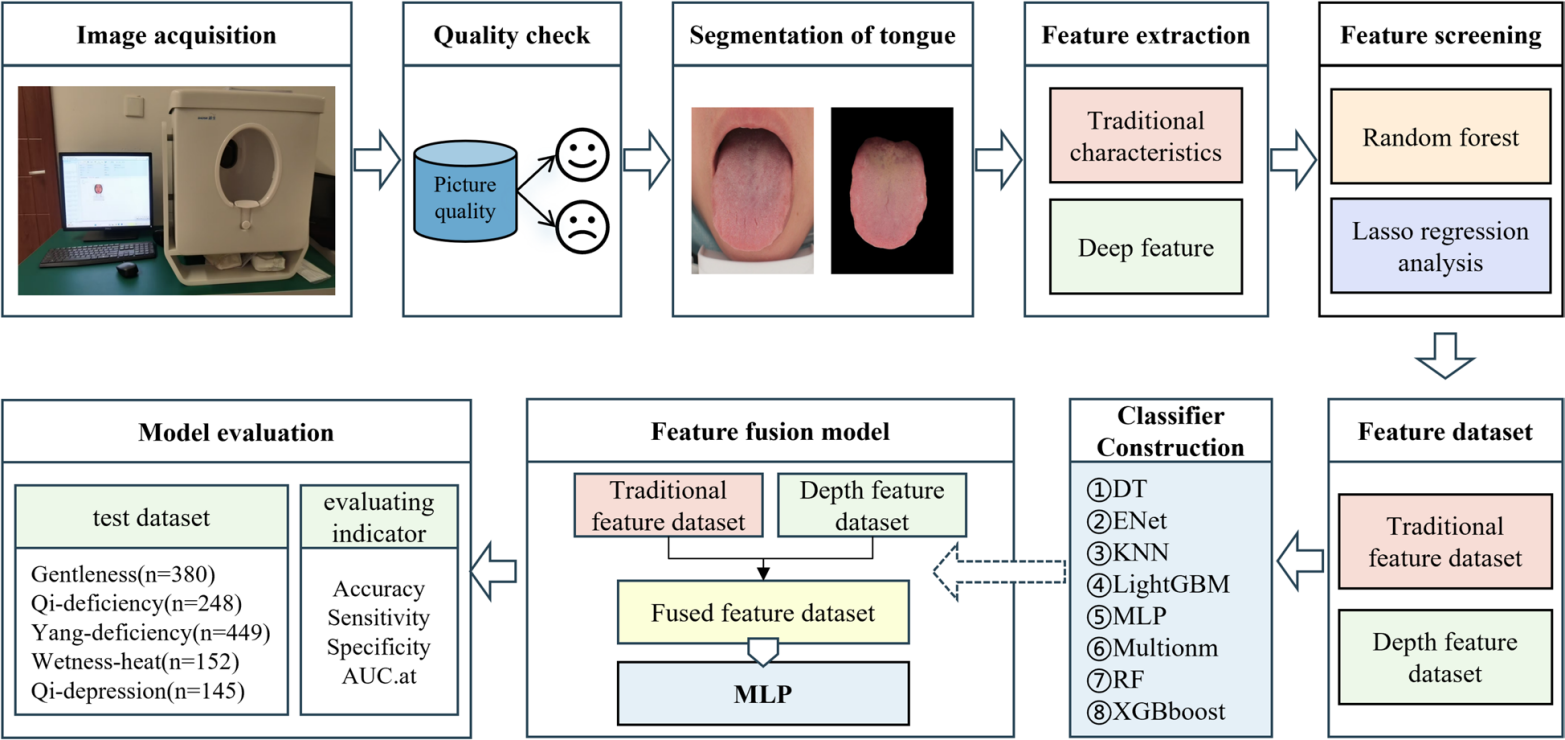
Flowchart of TCM Constitution identification model experiment

According to Wang Qi in a multi-centre, large-scale survey study covering a sample of 108,015 cases, among the many body types [16], gentleness (balanced) constitution type accounted for 28.98% of the total sample, while the imbalanced constitution was dominated by wetness-heat type (14.8%), qi-deficiency type (13.1%), and yang-deficiency type (11.2%) in men, and yang-deficiency type (20.0%), qi-deficiency type (13.2%), and qi-depression type (10.8%) were the main types in women. Taken together, the five constitution types, namely, gentleness-type, qi-deficiency type, yang-deficiency type, wetness-heat type, and qi-depression type, accounted for more than 80% of the overall samples, indicating that they occupy an important position in TCM constitutions. In view of this, this study aims to explore and construct an intelligent TCM constitution recognition classification model based on tongue pictures through in-depth analyses of the tongue features of people with the above five constitution types. The model is expected to provide more scientific and accurate support for health management and disease prevention related to TCM body composition. Figure 1 illustrates the overall design of the work.

## Materials and methods

### Data sources

The data for this study were obtained from subjects recruited from Dongzhimen Hospital affiliated with Beijing University of Traditional Chinese Medicine and Guang’anmen Hospital of China Academy of Traditional Chinese Medicine from 30 July 2023 to 30 August 2024, with data collected from a total of 1629 cases, which were screened by the inclusion and exclusion criteria, and finally included 380 cases of gentleness, 248 cases of qi-deficiency, 449 cases of yang-deficiency, 152 cases of wetness-heat, and qi-depression 145 cases (total 1374 cases). The study protocol and related documents were approved by the Medical Ethics Committee (Ethics Approval No. 2023DZMEC-228-01), and written informed consent was obtained from all subjects.

### Inclusion criteria

Inclusion Criteria were as follows. (a) Male and female, aged 18–65 years old; (b) no obvious history of acute and chronic diseases and critical illnesses within 3 months; (c) fully understand the content of the subject signed the informed consent form, and voluntarily conduct the physical test and tongue image collection; (d) good compliance, clear thinking, normal understanding and answer questions; (e) The result of the constitution type identification was one of gentleness, qi-deficiency, yang-deficiency, wetness-heat, and qi-depression.

### Exclusion criteria

**Fig. 2:**
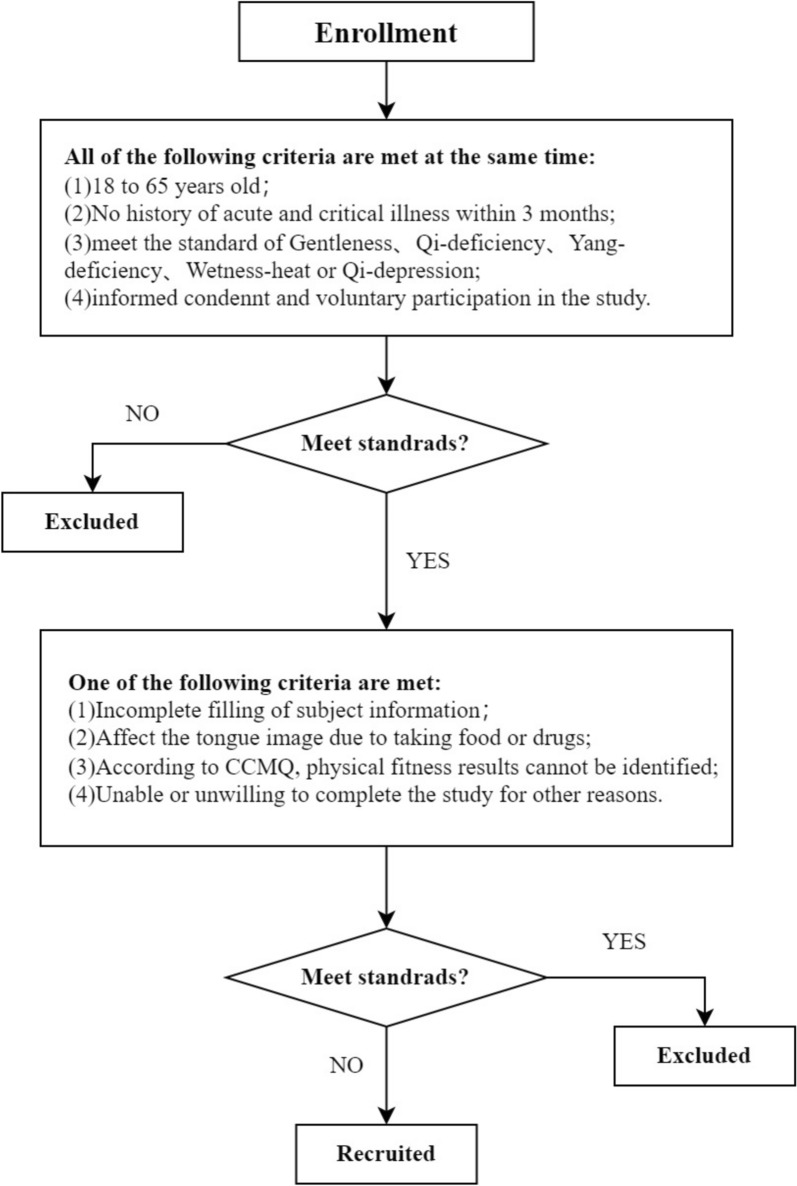
Flowchart of experimental inclusion and exclusion criteria

Exclusion Criteria were as follows. (a) Incomplete information data; (b) Taking food or medication affects the tongue image; (c) The results of the constitution could not be recognized according to the criteria for determining constitution; (d) Could not or did not want to complete the study due to other reasons. The flow chart of inclusion and exclusion criteria is shown in Fig. 2.

### TCM constitution criteria

Subjects were required to complete the constitution questionnaire in the questioning module of the Four Diagnostic Instrument (Model DS01-B, HUMC Note 20202200062) based on their health status in the last 3 months. The questionnaire was developed with reference to the Classification and Determination Table of Constitution in Traditional Chinese Medicine issued by the Chinese Society of Traditional Chinese Medicine in 2009. Subjects had to answer all the questions in the questionnaire and the transformed scores of the 9 subscales were calculated. Raw score = sum of the individual entry scores. Transformation score = [(raw score − number of entries)/(number of entries × 4)] × 100, with a score range of 0–100. If the transformation score of the peaceful constitution is ≥ 60 and the transformation scores of the other 8 constitutions are < 30, the constitution will be judged as balanced constitution. On the contrary, if the transformation scores of the other 8 somatic qualities were ≥ 40, they were judged as imbalanced.

### Criteria for tongue collection

**Fig. 3:**
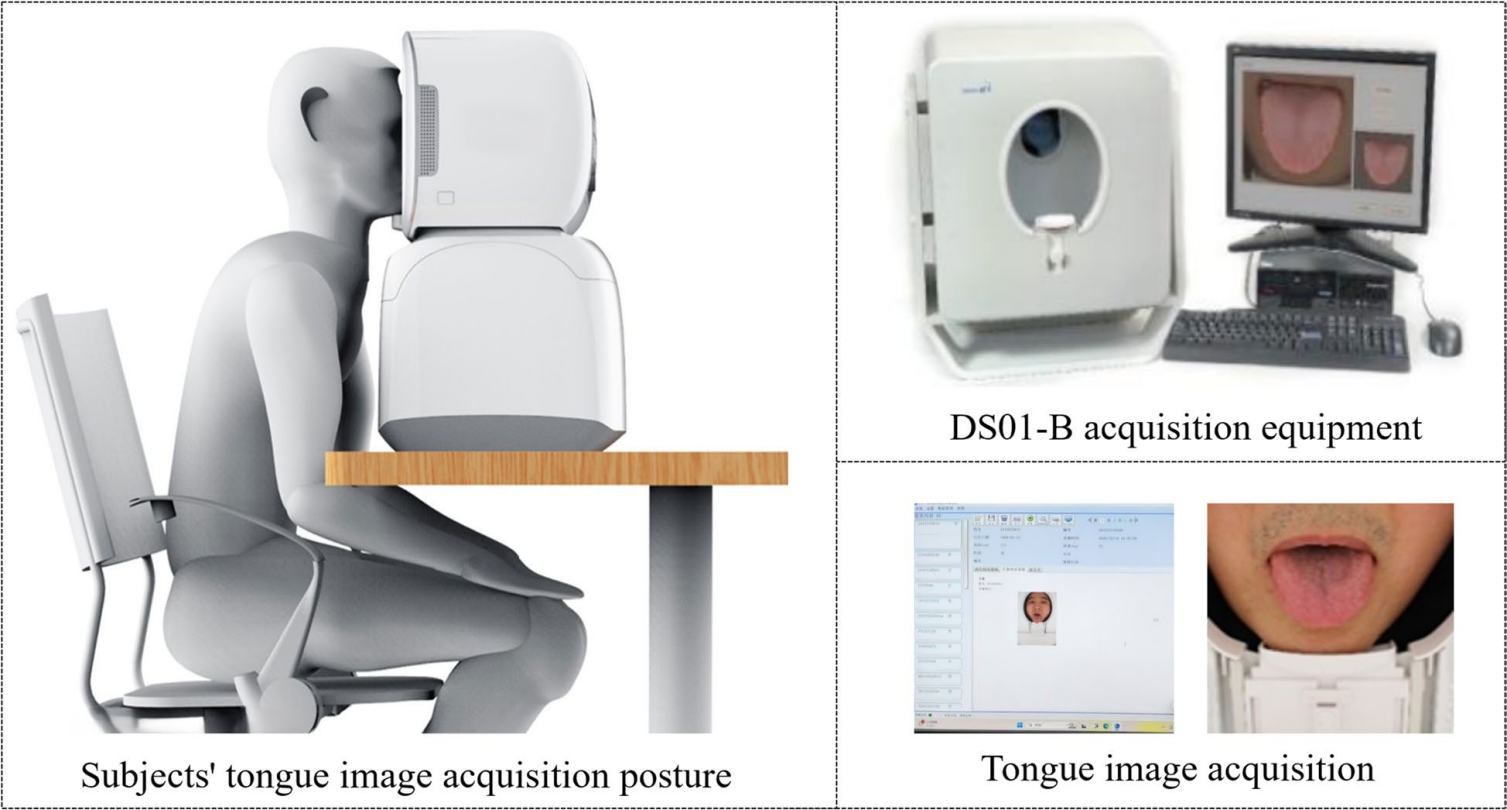
Device for tongue image data acquisition and TCM Constitution data acquisition

In this study, the acquisition of tongue data was conducted using the tongue diagnosis information collection module within the Four Diagnostic Instruments (DS01-B) system (Fig. 3). The tongue image collection chamber was constructed from a material with high light-occlusion properties and was designed in a box shape. It featured an oval opening on the front, allowing the participant’s face to be fully placed within the chamber while minimising the intrusion of external light sources. This setup created a closed and stable photographic environment, free from interference by external lighting and background distractions.

The tongue images were captured using a Canon 1200D camera, equipped with an APS-C format image sensor capable of capturing high-resolution images of up to 18 million pixels. To ensure accurate colour reproduction, an LED cold light source with a colour temperature of 6500 K was employed, with a shutter speed set at 1/200 s and an aperture of f/5.6 to maintain a moderate blur in the background, thus emphasising the subject. The ISO sensitivity was maintained at 200. The images captured by the camera were true to life and provided a detailed and accurate representation of the tongue, with dimensions of 1080 × 1920 pixels in full HD resolution, making them suitable for applications such as medical imaging that require high image quality.

Tongue images of all subjects were taken by a trained researcher operating the equipment. Prior to the acquisition of tongue images, the researcher ensured that the tongue was not stained, that there were no food residues on the tongue surface, and that the subject had not eaten for half an hour. After these conditions were met, the tongue images could be acquired. During the acquisition process, subjects were instructed to sit squarely in front of the tongue diagnostic apparatus, with the lower jaw placed on the jaw rest of the quadruple diagnostic apparatus, and to open the mouth as wide as possible, sticking out the tongue, keeping the tongue body relaxed, the tongue surface spread out, and the tip of the tongue slightly drooping. Tongue images were captured twice for each subject and the higher quality images were retained for study analysis.

### *UNet*++* improved networks for image segmentation*

**Fig. 4:**
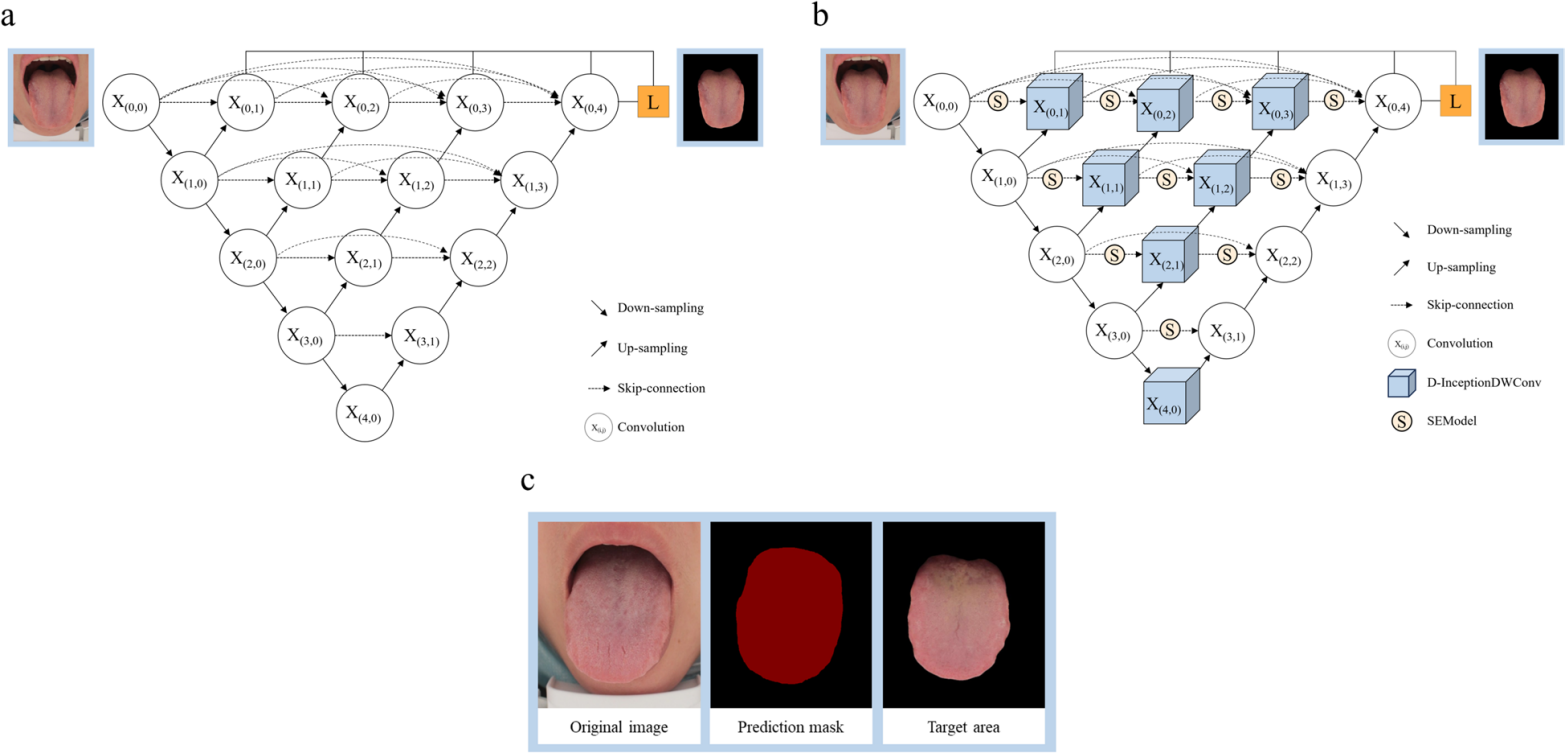
Tongue image segmentation model architecture and segmentation results example. **a** Overview of the architecture of UNet++. **b** Overview of the architecture of ISE-UNet++. **c** Example of tongue image segmentation results

The original image acquired for the experiment contains the complete tongue information as well as some surrounding skin. To avoid the interference of irrelevant regions, we segmented the tongue target region from the original image. In order to accurately segment the tongue image, we used a modified UNet++ model (Fig. 4a) for the segmentation of the tongue target region to exclude the interference of irrelevant background information [17].

U-Net++ is a further optimization and extension of the U-Net model [18]. The model realizes a direct connection between the layers of the encoder and the decoder by redesigning the jump path, which enables the features of each layer to be passed to all subsequent layers of the decoder, effectively preserving the multi-scale feature information of the image. Compared with the traditional U-Net model, U-Net++ also incorporates a deep supervision technique, which dynamically adjusts the weights of the feature map, enhances the focus on key features, and accurately extracts important information from the image.

Using ISE-UNet++ (Fig. 4b) for tongue segmentation, the ISE-UNet++ model replaces the convolutional layer in the middle of the UNet++ model with an extraction layer composed of two InceptionDWConv2 d in tandem [19], and at the same time adds SE (Squeeze-and-Excitation) attention between the convolution in the middle of the model mechanism [20]. Compared with the UNet++ model, ISE-UNet++ can integrate multi-scale information more fully by connecting two InceptionDWConv2 d in tandem, and capture the features of tongue particles, tongue texture, and the overall contour of the tongue through different sizes of convolution kernels, and also reduce the number of parameters effectively through the depth-separable convolutions to improve the computational efficiency and significantly enhances the anti-interference ability and generalisation ability of the model, enabling it to segment the tongue image more stably and adapt to the differences between individuals. In addition, the SE attention mechanism can reweight the feature channels so that the model can adaptively enhance the important features and suppress the unimportant features, which improves the model's representational ability, and at the same time, enhances the anti-interference ability and improves the generalisation ability in terms of enhancing the robustness of the model.

### Tongue image feature extraction

#### Traditional feature extraction

Tongue color features were extracted using Lab color space, which is defined by three parameters: luminance (L*), green to red (a*) and blue to yellow (b*). It is designed based on human visual perception with perceptual homogeneity, which makes changes in the values of the parameters produce similar changes visually. Lab color space is more intuitive and convenient for color adjustment than color spaces such as RGB and CMYK [21].

In this study, we used Grey Level Co-occurrence Matrix (GLCM) to extract the texture features of the tongue image [22]. GLCM is a widely used statistical method in image processing to quantify the spatial correlation of pixel grey values in an image to describe the texture properties of the image. It captures the combined information of an image in different directions, intervals, and magnitude of grey scale changes by calculating the frequency of co-occurrence of grey scale values between any two pixels in an image separated by a specific distance and direction. GLCM is able to reflect the characteristics of the image texture in terms of roughness, contrast, uniformity and correlation, which provides a rich quantitative description for subsequent analysis.

The tongue color, tongue shape, tongue coating color and coating texture features on the tongue image were annotated by three TCM practitioners with 20 years of clinical experience in the profession with the title of deputy director or above. When two of them agreed, the annotated image was adopted; when they disagreed, the third physician joined in judging the annotation, and the three of them decided the annotation result after discussion. Fleiss’ Kappa is a statistical measure used to assess the agreement among multiple raters, particularly suitable for evaluation systems involving several independent classifiers [23]. To further evaluate the consistency of annotations made by three physicians, we calculated the Fleiss'Kappa coefficient. The results indicated that the coefficients for tongue colour, tongue shape, coating colour, and coating texture were 0.79, 0.81, 0.76, and 0.78, respectively, suggesting a strong level of agreement among the three physicians (with Kappa coefficients > 0.75 indicating high consistency). This finding underscores that, despite the inherent subjectivity in traditional Chinese medicine diagnosis, the data annotations in this study exhibit good reliability and consistency, attributable to the stringent selection of experts and a rational decision-making framework.

#### Deep feature extraction

**Fig. 5:**
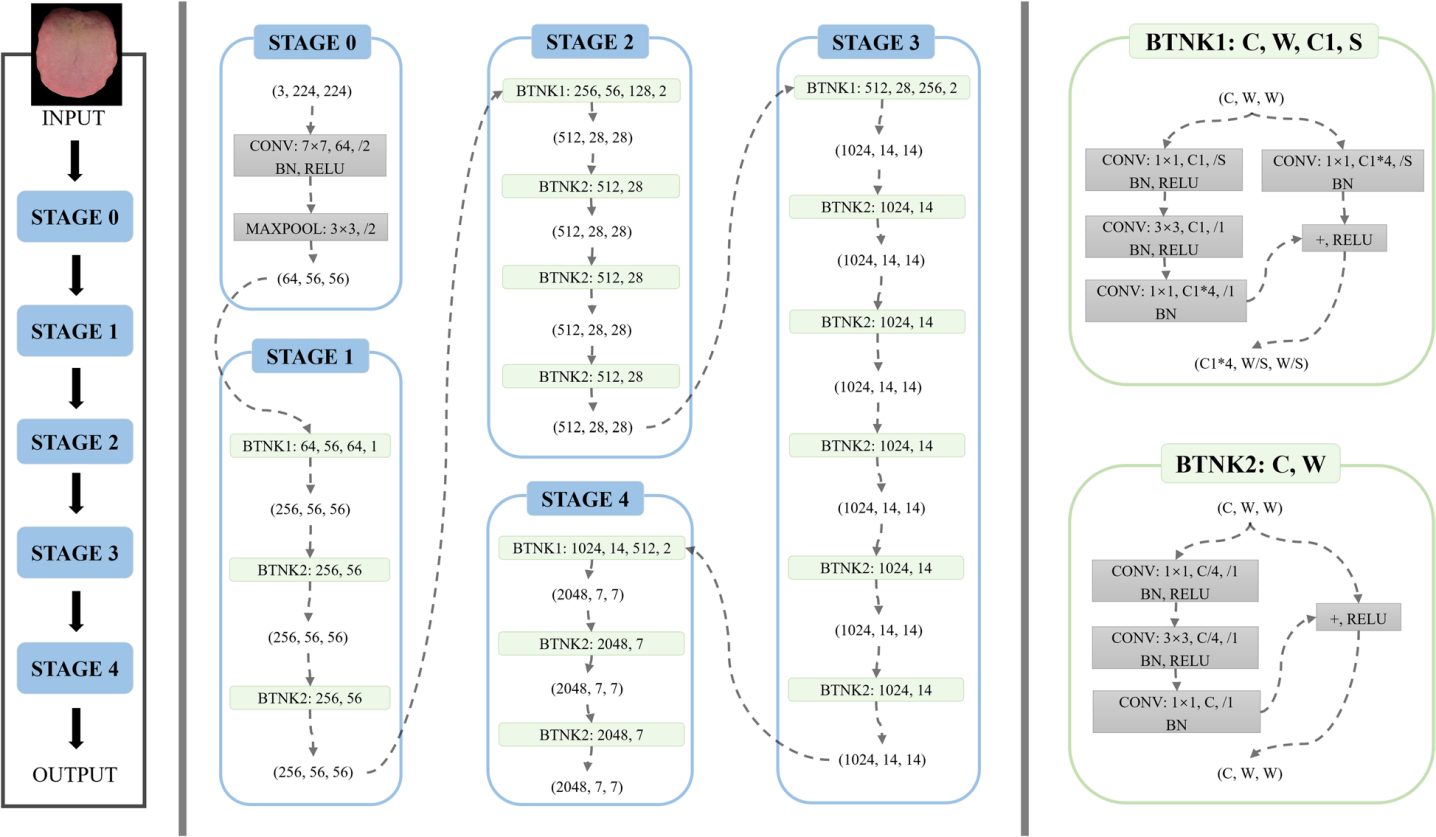
ResNet50 deep feature extraction network

In this study, we selected ResNet-50 as the core architecture for deep feature extraction of tongue images (as shown in Fig. 5), primarily due to its ability to address the degradation problem in deep networks, its efficient feature representation, and its strong transfer learning capabilities [24, 25]. Compared to VGG, ResNet-50 achieves higher accuracy with fewer parameters while effectively mitigating the risk of overfitting [26]. In contrast to EfficientNet, which optimises the network structure through a compound scaling method, ResNet-50 has not demonstrated significant superiority in practical applications, particularly in the field of medical image analysis. Moreover, the increased complexity of EfficientNet may extend training times and complicate hyperparameter tuning [27]. Therefore, considering the balance between performance and resource utilisation, along with support from existing literature, we determined ResNet-50 to be the most suitable choice after a comprehensive evaluation of model performance, computational efficiency, and practical application requirements. This decision enables efficient extraction of deep features while ensuring robust generalisation capability. Future research will further explore the potential of other advanced network architectures in the context of constitution identification in traditional Chinese medicine.

First, we utilized U-Net++ for precise segmentation of tongue images. The segmented images were subsequently used to construct the training dataset. Before training, the network parameters were initialised by loading the pre-training weights of the model on the ImageNet dataset. During training, we set the learning rate to 1 × 10^–3^, used Stochastic Gradient Descent (SDG) as the optimisation algorithm, and chose categorical cross-entropy as the loss function [28]. When the model reaches a converged state, the output of its penultimate layer is extracted as a deep feature representation. These deep features exist in the form of a has-been tensor containing 2048 elements, which provides rich depth information for subsequent analyses.

### Screening of tongue features

#### Data preprocessing

Data preprocessing improves data quality and consistency by cleaning and standardizing the data, thus improving the accuracy of the analysis results and model performance.

Specifically, both color features and texture features were processed using standardization methods. This step is done with the help of StandardScaler [29], which aims to adjust the mean value of each feature to 0 and its standard deviation to 1. The aim is to eliminate the differences in scale between different features, thus improving the efficiency and stability of model training.

For unordered category variables, we use the OneHotEncoder method [30]. This method converts category variables into a series of binary columns, each representing a specific category value. Through this conversion, the category variables can be efficiently processed by machine learning algorithms while effectively avoiding the potential impact of category order on model performance.

In this study, we examined the binary variables in the dataset to ensure that their data types were integer or Boolean and that each variable had values of 0 or 1 only [31]. Meanwhile, we examined the binary variables for missing values and outliers, and interpolated them using plurals to ensure data integrity. For outliers, we took measures such as replacing them with default values or performing data audits.

After completing the data preprocessing, we integrate all the preprocessed features into a unified data framework, which prepares a complete dataset for subsequent model training and prediction.

#### Screening for important features of the tongue

In Chinese medicine diagnostics, tongue image, as an important part of diagnosis, contains rich physiological and pathological information [32]. In order to extract the features that have a significant effect on the target variables from the complex tongue image data, this study proposes to adopt two machine learning methods, Lasso regression analysis and random forest, to screen the traditional features and deep features of tongue image, and construct a prediction model based on the screened features.

Lasso regression analysis is a machine learning algorithm that combines regression analysis with feature selection [33]. It achieves feature selection and dimensionality reduction by introducing an L1 regularisation term, which makes some of the regression coefficients compressed to zero. In tongue image feature selection, Lasso regression analysis can effectively identify features that are highly correlated with the target variable, while excluding those that contribute less to the prediction results.

Random forest (RF) is an integrated learning method based on decision trees, which improves prediction accuracy by constructing multiple decision trees and combining their outputs [34]. In terms of feature selection, Random Forest is able to assess the importance of each feature in the model building process. This is usually done by calculating the Gini index of the information gain or loss brought about by the feature when the node splits. In tongue feature screening, Random Forest is able to identify those features that are important for the classification results, such as the morphology of the tongue body and the thickness of the tongue coating. In addition, Random Forest can handle high dimensional data and missing data, making it widely applicable in practical applications.

Lasso regression analysis and RF have their own advantages in feature screening. Lasso regression analysis is good at dealing with linear relationships and feature selection, while RF is able to capture non-linear relationships and interactions between features. To extract features from complex tongue image data that significantly impact the target variable, this study employed two machine learning methods—Lasso regression analysis and Random Forest—for feature selection, subsequently constructing predictive models based on the selected features. In the Lasso regression, the optimal value of the λ parameter was automatically selected through tenfold cross-validation (cv = 10) to minimize the mean squared error (MSE) as the selection criterion. For feature selection using Random Forest, the maximum number of trees (n_estimators) was set to 500 and the maximum depth (max_depth) to 15, with the Gini index used as the splitting criterion. Feature importance was ranked based on the average decrease in Gini index, combined with the intersection results to ensure the stability of the selected features.

#### Sample balancing strategy

Considering the issue of class imbalance in the dataset, we employed targeted strategies, including a weighted loss function and SMOTE (Synthetic Minority Over-sampling Technique), to address this challenge. When training the deep learning model, we used a weighted cross-entropy loss function based on class frequencies. The weight for each class is inversely proportional to the number of samples in that category [35]. For the underrepresented “Wetness-heat” condition (n = 152), its weight is significantly higher than that of the more prevalent “Moderate” condition (n = 380). This approach allows for greater emphasis on minority classes during the training process, effectively mitigating biases caused by class imbalance. In addition to adjusting the loss function, we also applied SMOTE to generate synthetic samples for the minority class [36]. This methodology increased the number of minority class samples in the dataset, enabling the model to better recognize the features of all classes during the learning process, thereby enhancing classification accuracy.

### Model establishment and validation

#### Model construction

We take the filtered tongue features and divide them into 3 groups of labels, which are tongue traditional feature group, tongue deep feature group and tongue fusion feature group. Secondly, we choose eight machine learning algorithms, namely decision tree (DT), random forest (RF), K-nearest neighbour (KNN), unordered multiclassified logistic regression analysis (Multionm), ElasticNet (Enet), multilayer perceptual machine (MLP), LightGBM, and XGBboost, to construct an intelligent identification model of TCM constitution based on the tongue image respectively.

The use of Accuracy, Sensitivity, Specificity, AUC, Recall, F1, MCC, KAP, etc. was adopted to evaluate the performance of the model [37]. The total sample N, True Positive (TP) denotes the number of samples in which the model predicts a positive class and is actually positive, True Negative (TN) denotes the number of samples in which the model predicts a negative class and is actually negative, False Positive (FP) denotes the number of samples in which the model predicts a positive class but is actually negative, and False Negative (FN) denotes the number of samples where the model predicts a negative class but is actually a positive class.

1 $$Accuracy = \frac{TP + TN}{{TP + TN + FP + FN}}$$

2 $$Precision = \frac{TP}{{TP + FP}}$$

3 $$Sensitivity/Recall = \frac{TP}{{TP + FN}}$$

4 $$Specificity = \frac{TN}{{TN + FP}}$$

5 $$F1 = 2 \times \frac{Precision \times Recall}{{Precision + Recall}}$$

6 $$Precision = \frac{TP}{{TP + FP}}$$

7 $${\text{MCC}} = \frac{TP \times TN - FP \times FN}{{\sqrt {\left( {TP + FP} \right)\left( {TP + FN} \right)\left( {TN + FP} \right)\left( {TN + FN} \right)} }}$$

8 $$KAP = \frac{{\left( {\frac{TP + TN}{N}} \right) - \left( {\frac{{\left( {TP + FP} \right)\left( {TP + FN} \right) + \left( {TN + FP} \right)\left( {TN + FN} \right)}}{{N^{2} }}} \right)}}{{1 - \left( {\frac{{\left( {TP + FP} \right)\left( {TP + FN} \right) + \left( {TN + FP} \right)\left( {TN + FN} \right)}}{{N^{2} }}} \right)}}$$

9 $$MIOU = \frac{TP}{{FN + TP + FP}}$$

10 $$MPA = \frac{TP + TN}{{FN + TP + FP + TN}}$$

#### Model validation

In order to evaluate the performance of the constructed model and optimise its parameters, we performed a rigorous internal validation of the tongue-somatic dataset [38]. We randomly divided the dataset into a training set and a validation set in a ratio of 8:2, where 80% of the data were used for model training and 20% for initial validation of the model's performance. To further ensure the stability and generalisation ability of the model, we perform fivefold Cross-Validation, i.e., the original dataset is divided into five subsets, four subsets are used as the training set and one subset is used as the validation set in turn, and the results of the five validations are finally aggregated to obtain a more reliable and representative performance evaluation index. Through this comprehensive validation strategy, we can not only effectively prevent model overfitting, but also more comprehensively assess the performance of the model on unseen data, thus providing a more scientific and reliable tool for TCM constitution identification.

## Results

### Basic information on included subjects

The baseline characteristics of the study population are summarised in Table 1. Overall, the results of the age distribution among the different constitution traits showed that the mean ages of gentleness, qi-deficiency, yang-deficiency, wetness-heat, and qi-depression were 50 ± 12, 50 ± 12, 50 ± 10, 48 ± 10, and 46 ± 11, respectively. Although the age data for males and females did not satisfy a normal distribution, their variances were chi-square. The Mann–Whitney U test revealed that there was no significant difference between the age distributions of males and females at a significance level of 0.05.

**Table 1:** Baseline characteristics of the study cohort

Characteristic	Training and validation dataset^a^					p-value^b^
	Gentleness N = 380	Qi-deficiency N = 248	Yang-deficiency N = 449	Wetness-heat N = 152	Qi-depression N = 145	
Age (years)	50 (12)	50 (12)	50 (10)	48 (10)	46 (11)	0.0818
Sex (female/male)	161/219	139/109	295/154	46/106	109/36	< 0.001

^a^Mean (SD); n (%)

^b^Shapiro-Wilk Test; Levene’s Test; Mann–Whitney U Test; Chi-square Test

We assessed the association between gender and five body constitution types using the Chi-squared test. The results indicated a significant correlation between gender and body constitution types overall (p < 0.0001), suggesting that gender may be an important factor influencing body constitution classification. However, considering the potential risk of Type I error when testing multiple body constitution types, we applied the Bonferroni and Holm–Bonferroni correction methods to adjust the p-values. After correction, the differences in gender distribution for each specific constitution type did not reach statistical significance (adjusted p-values were all 1.0000). This outcome suggests that while there is a significant overall association between gender and body constitution types, the gender differences within individual constitution types are not pronounced when accounting for multiple comparison adjustments.

Nevertheless, in light of the potential significant impact of gender on body constitution types observed in clinical practice, and to minimize confounding factors, we included gender as a control variable in our model construction. Gender was converted into a binary variable (0 for male and 1 for female) and incorporated into all models involving body constitution classification. This approach aims to reduce potential biases arising from sample imbalance and improve the model's generalization ability and predictive performance.

### Segmentation results of tongue image

**Fig. 6:**
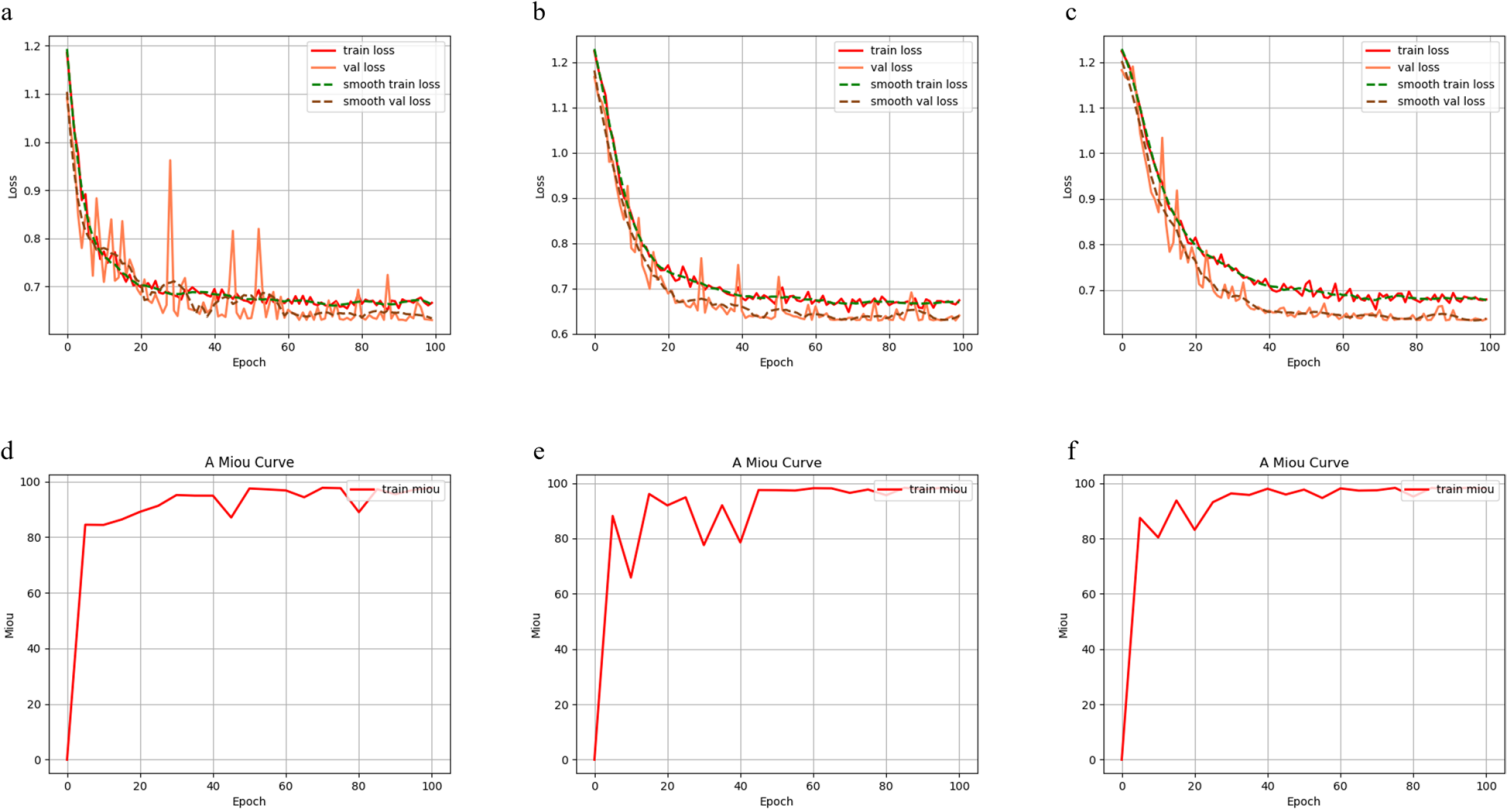
MIoU and loss curves of the model training process. **a** Unet model training loss curve. **b** Unet++ model training loss curve. **c** ISE-UNet++ model training loss curve. **d** Unet model Miou curve. **e** Unet++ model training Miou curve. **f** ISE-UNet++ model training Miou curve

In the segmentation task targeting tongue images, we used MIoU, MPA and Accuracy as evaluation metrics to compare the segmentation performance of ISE-Unet++ with the original UNet++, UNet. As shown in Fig 6, the experimental results show that ISE-UNet++ achieves a certain improvement in segmentation effect compared to the original UNet++, UNet. Specifically, in the segmentation of tongue image, ISE-UNet++ improves MIoU by 0.72%, MPA by 1.31%, and Accuracy by 0.91% compared to UNet algorithm; and improves MIoU by 0.54%, MPA by 1.02%, and Accuracy by 0.86% compared to UNet++ algorithm (Table 2). This result shows that ISE-UNet++ not only effectively improves the segmentation accuracy, but also significantly reduces the loss of edge information, which can better meet the segmentation needs of tongue images.

**Table 2:** Validation set results of the ISE-UNet++ and UNet++

Model	MIOU	MPA	Accuracy
Tongue_UNet	97.73	97.95	98.26
Tongue_UNet++	97.91	98.24	98.31
Tongue_ISE-UNet++	98.45	99.26	99.17

### Screening results for traditional features of the tongue

**Fig. 7:**
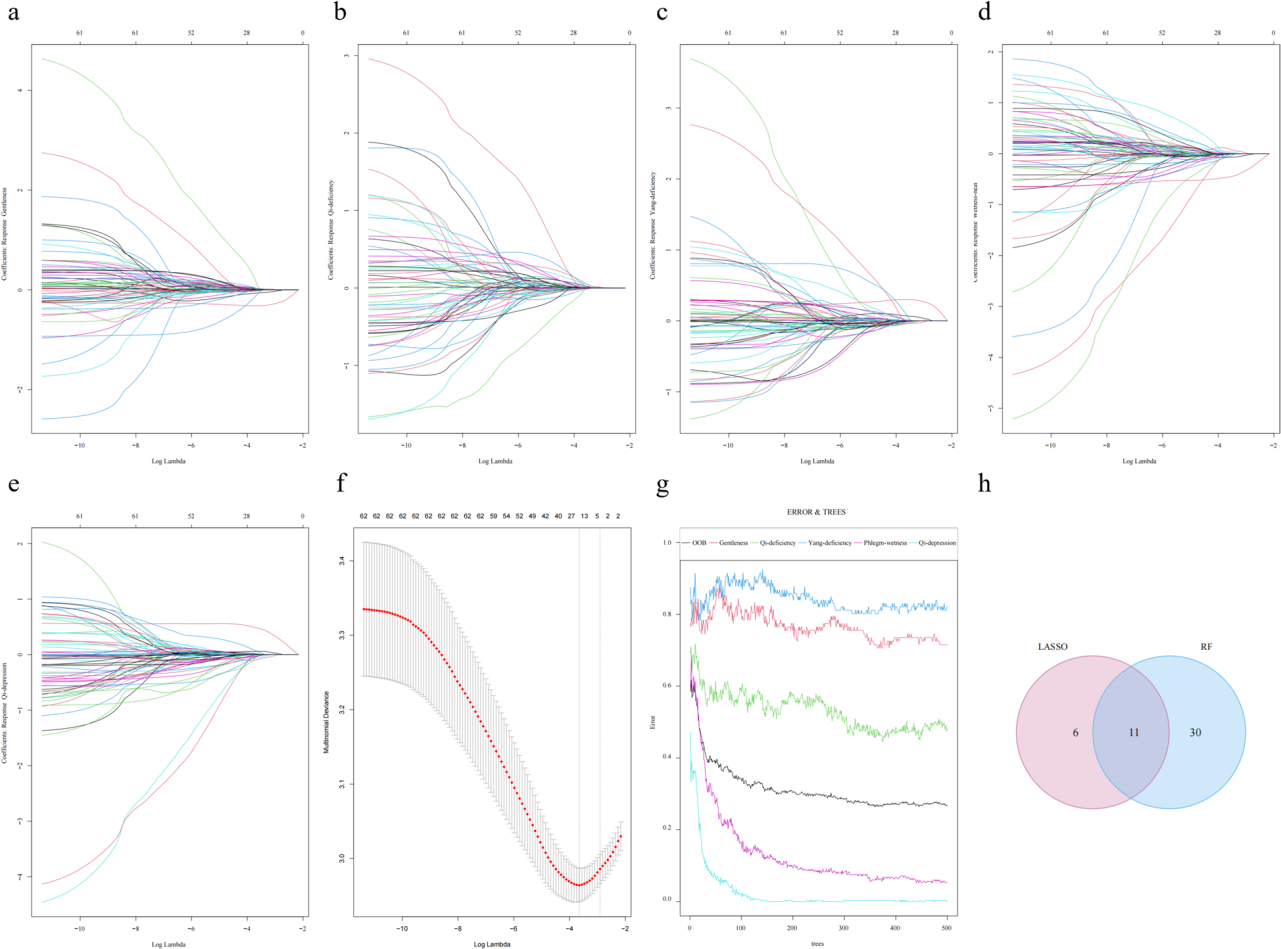
LASSO analysis and RF for selecting traditional features. **a** Lasso analysis of Gentleness. **b** Lasso analysis of Qi-deficiency. **c** Lasso analysis of Yang-deficiency. **d** Lasso analysis of Wetness-heat. **e** Lasso analysis of Qi-depression. **f** Feature screening of Lasso analysis. **g** Feature screening of RF. **h** Wayne diagram of feature selection intersection between RF and Lasso analysis

Figure 7 illustrates the detailed results of the LASSO regression analysis with the Random Forest tongue traditional feature screening. Specifically, by adjusting the logarithmic transformation (Log Lambda) of the regularisation parameter λ for the Lasso regression analysis, we observed trends in the coefficients of the different features to determine which features were most important for model prediction. The experimental results show (Fig. 7f) that as λ decreases (i.e., Log Lambda increases), the coefficients of most of the features gradually decrease and eventually become zero, indicating that these features are phased out of the model, while the coefficients of a few features maintain larger values or change less throughout the process, indicating that they are important for model prediction. Lasso regression is performed through the introducing the L1-paradigm penalty term, which enables simultaneous feature selection and parameter estimation, thus effectively identifying 17 statistically significant feature variables whose regression coefficients in the model are significantly non-zero, suggesting that they have significant predictive power for body mass classification.

At the same time, we also used the RF algorithm to rank the importance of the tongue features, and in this way, we filtered out the traditional features of the tongue that had the most influence on the classification results. The experimental results show that in the constructed multi-classification model, the error rates of different types show different trends with the increase of the number of trees (Fig. 7g). The overall error rate (OOB) gradually decreases with the growth of the number of trees, indicating improved model performance. Specifically for each category, there is a significant decrease in the error rate for the gentleness, qi-deficiency and wetness-heat type, especially the gentleness type is the most obvious; this suggests that for these constitution categories, RF is able to efficiently extract useful information from the tongue features, thus improving the classification accuracy. In addition, by constructing multiple decision trees and evaluating the importance of the features, 41 key features were screened out, which played a key role in the node splitting of the model and had high feature importance scores.

To further validate the robustness and reproducibility of these features, we used Lasso regression for feature selection and performed an intersection analysis with the results of random forest (Fig. 7h). By taking the intersection of Lasso regression and random forest screening results, we finally identified 11 (11 < 17 and 11 < 41) robust and well reproducible features. These features showed high discriminative power and stability in discriminating different TCM body types, providing a solid foundation for subsequent model construction.

### Screening results for deep feature s of the tongue

**Fig. 8:**
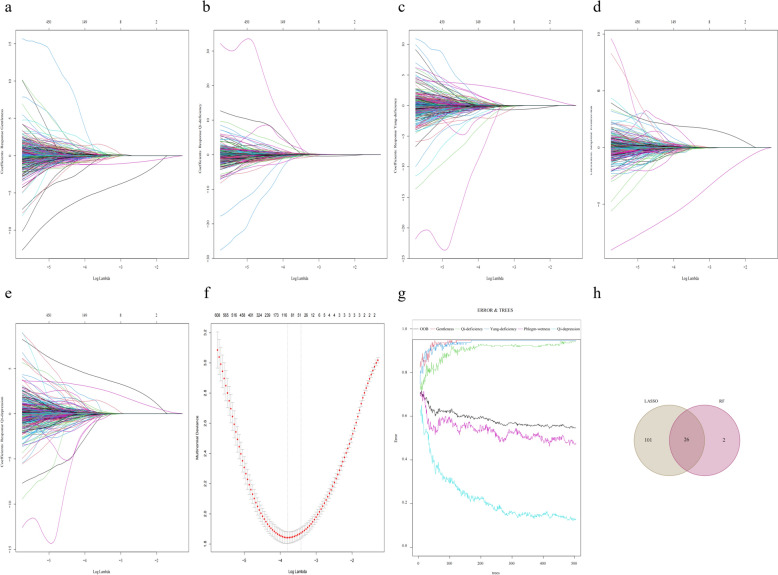
LASSO analysis and RF for selecting deep features. **a** Lasso analysis of Gentleness. **b** Lasso analysis of Qi-deficiency. **c** Lasso analysis of Yang-deficienc. **d** Lasso analysis of Wetness-heat. **e** Lasso analysis of Qi-depression. **f** Feature screening of Lasso analysis. **g** Feature screening of RF. **h** Wayne diagram of feature selection intersection between RF and Lasso analysis

Based on the 2048 deep features extracted in the previous stage we applied the Random Forest and Lasso regression models to screen the key features that have a significant effect on the target variables. The results show (Fig. 8) that through this combination of deep learning and traditional machine learning, we identified key deep features. This set of features not only contains subtle differences that are not easily noticeable visually, but also reflects the complex patterns in the tongue images. After cross-validation, the final number of selected deep features is 26, which show excellent performance in the prediction model and demonstrate unique value in distinguishing different TCM body types. In particular, we found that certain deep features, although not prominent in the traditional feature space, are highly discriminative in the deep feature space, which provides new perspectives for understanding the relationship between tongue images and TCM body types.

### Results of models based on a single feature of tongue image

In this study, eight different machine learning algorithms, namely Decision Tree (DT), Elastic Net (Enet), K-Nearest Neighbors (KNN), LightGBM, Multilayer Perceptron (MLP), Multiple Linear Regression (MLR), and XGBoost, were used to evaluate the potential application of traditional features and deep features in TCM constitution recognition. Regression (MLR), and XGBoost to evaluate the potential of traditional and deep features of tongue image in TCM constitution recognition. This approach aims to minimise potential bias due to specific model preferences through a wide range of model choices.

**Fig. 9:**
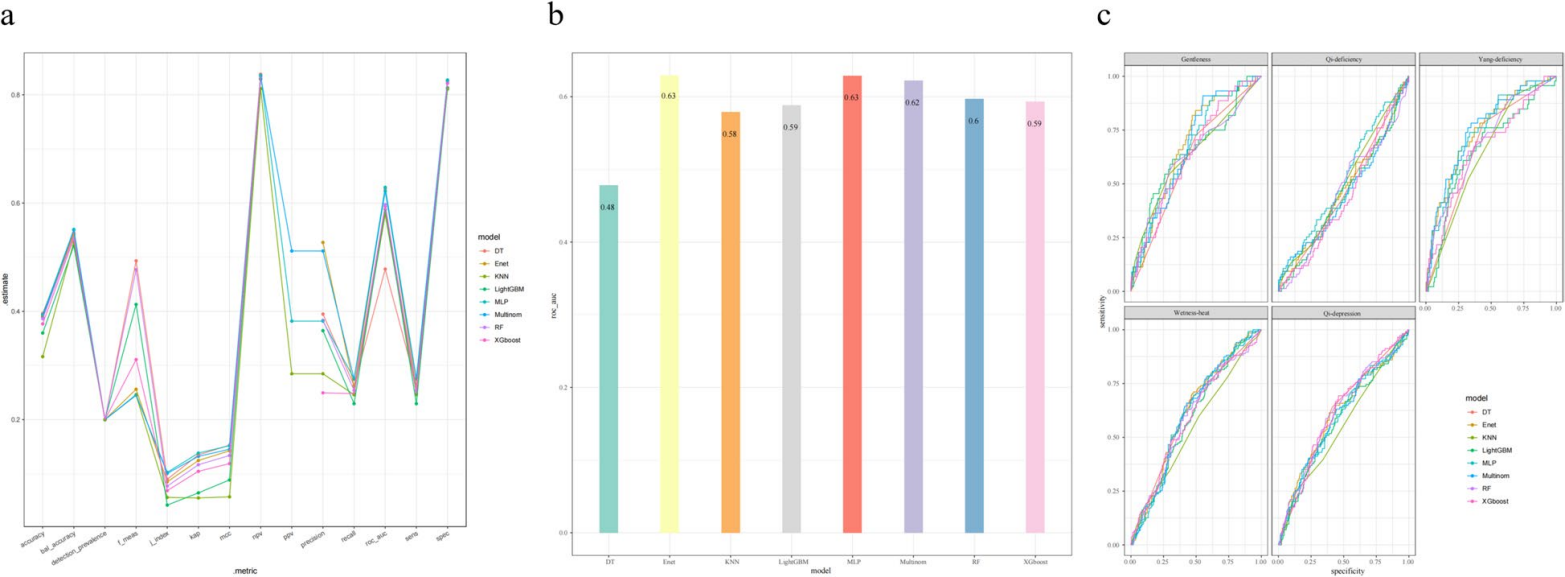
Model results based on traditional feature dataset of tongue image. **a** Broken line graph of model evaluation indicators. **b** AUC histogram of model. **c** ROC curve of model

**Fig. 10:**
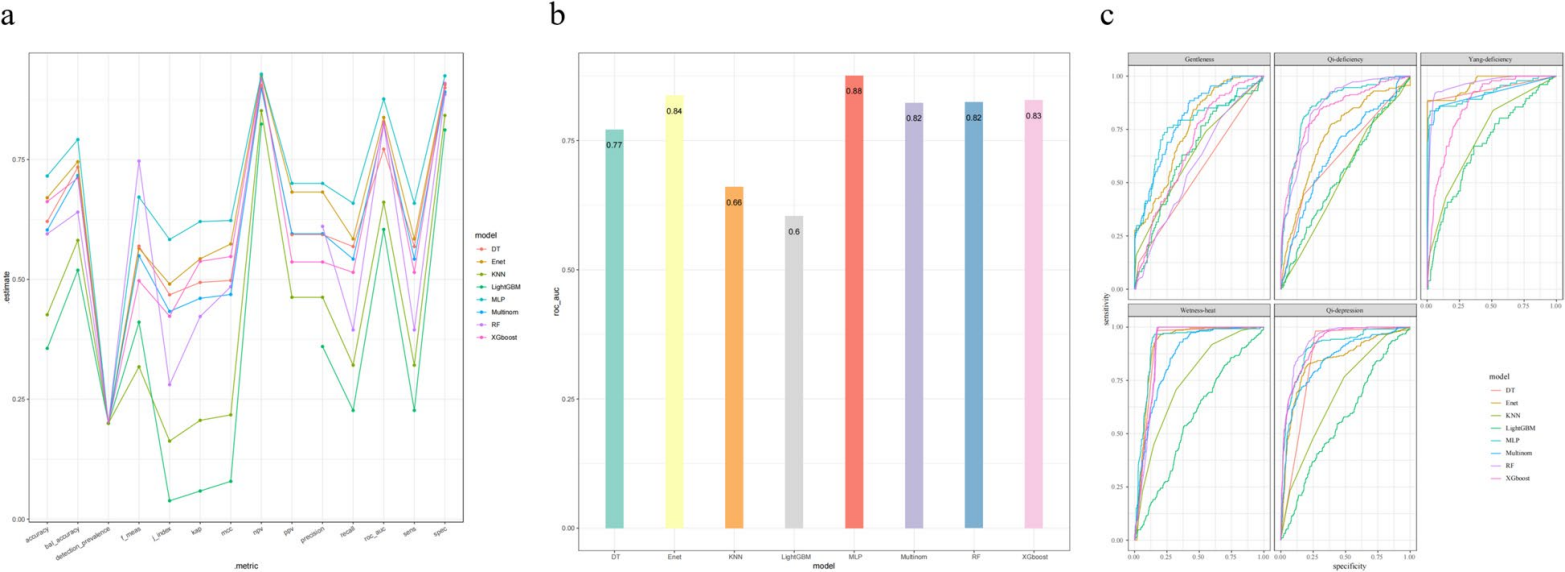
Model results based on deep feature dataset of tongue image. **a** Broken line graph of model evaluation indicators. **b** AUC histogram of model. **c** ROC curve of model

Tables 3 and 4 show the key performance metrics of the eight machine learning models based on single conventional and deep features of the tongue on the validation set. The results show that the MLP algorithm performs best in the model based on a single feature of the tongue. Specifically, in the traditional feature model (Fig. 9), the accuracy of MLP is 0.393, AUC is 0.629, sensitivity is 0.275, and specificity is 0.827; in the deep feature model (Fig. 10), the accuracy of MLP is significantly improved to 0.715, with an AUC of 0.876, a sensitivity of 0.658, and a specificity of 0.924. However, these values still do not fully meet the requirements of practical applications, indicating that relying on a single feature may not be sufficient to adequately capture the complex information required for TCM constitution recognition, suggesting that we need to further explore to improve the overall performance of the model through feature fusion and other methods.

**Table 3:** Evaluation index results of the model based on traditional features of tongue image

	Accuracy	Sensitivity	Specificity	F1	AUC	KAP	recall	npv	mcc
Multinom	0.389	0.275	0.826	0.246	0.622	0.132	0.275	0.834	0.145
DT	0.396	0.263	0.827	0.493	0.478	0.135	0.263	0.838	0.153
Enet	0.391	0.260	0.825	0.256	0.626	0.125	0.260	0.836	0.143
KNN	0.316	0.246	0.810	0.245	0.579	0.055	0.246	0.811	0.057
LightGBM	0.360	0.229	0.813	0.413	0.588	0.065	0.229	0.828	0.089
MLP	0.394	0.275	0.827	0.245	0.629	0.138	0.275	0.836	0.152
RF	0.386	0.254	0.823	0.477	0.597	0.117	0.254	0.834	0.134
XGboost	0.377	0.248	0.821	0.311	0.593	0.105	0.248	0.830	0.119

**Table 4:** Evaluation index results of the model based on deep features of tongue image

	Accuracy	Sensitivity	Specificity	F1	AUC	KAP	recall	npv	mcc
Multinom	0.603	0.542	0.891	0.549	0.823	0.460	0.542	0.898	0.468
DT	0.670	0.584	0.906	0.565	0.838	0.543	0.584	0.925	0.574
Enet	0.621	0.568	0.899	0.569	0.771	0.494	0.568	0.904	0.498
KNN	0.595	0.395	0.886	0.747	0.824	0.423	0.395	0.917	0.485
LightGBM	0.662	0.514	0.909	0.497	0.828	0.538	0.514	0.920	0.548
MLP	0.715	0.659	0.924	0.671	0.876	0.621	0.659	0.928	0.623
RF	0.356	0.227	0.812	0.411	0.604	0.059	0.227	0.824	0.079
XGboost	0.426	0.321	0.842	0.318	0.661	0.206	0.321	0.852	0.217

Despite the excellent performance of MLP in single-feature modelling, the overall classification effect of the TCM constitution recognition model based on a single tongue feature is still unsatisfactory. Therefore, we performed feature fusion based on the pre-screening results of traditional tongue features and depth features, and selected the MLP model with the best overall performance to construct a multi-classification model that fuses traditional tongue features and depth features. Through feature fusion, we expect to develop a more robust and accurate model, which will improve the effect of TCM constitution identification and enhance the accuracy of constitution identification.

### Results of a test set of MLP models based on tongue fusion features

**Fig. 11:**
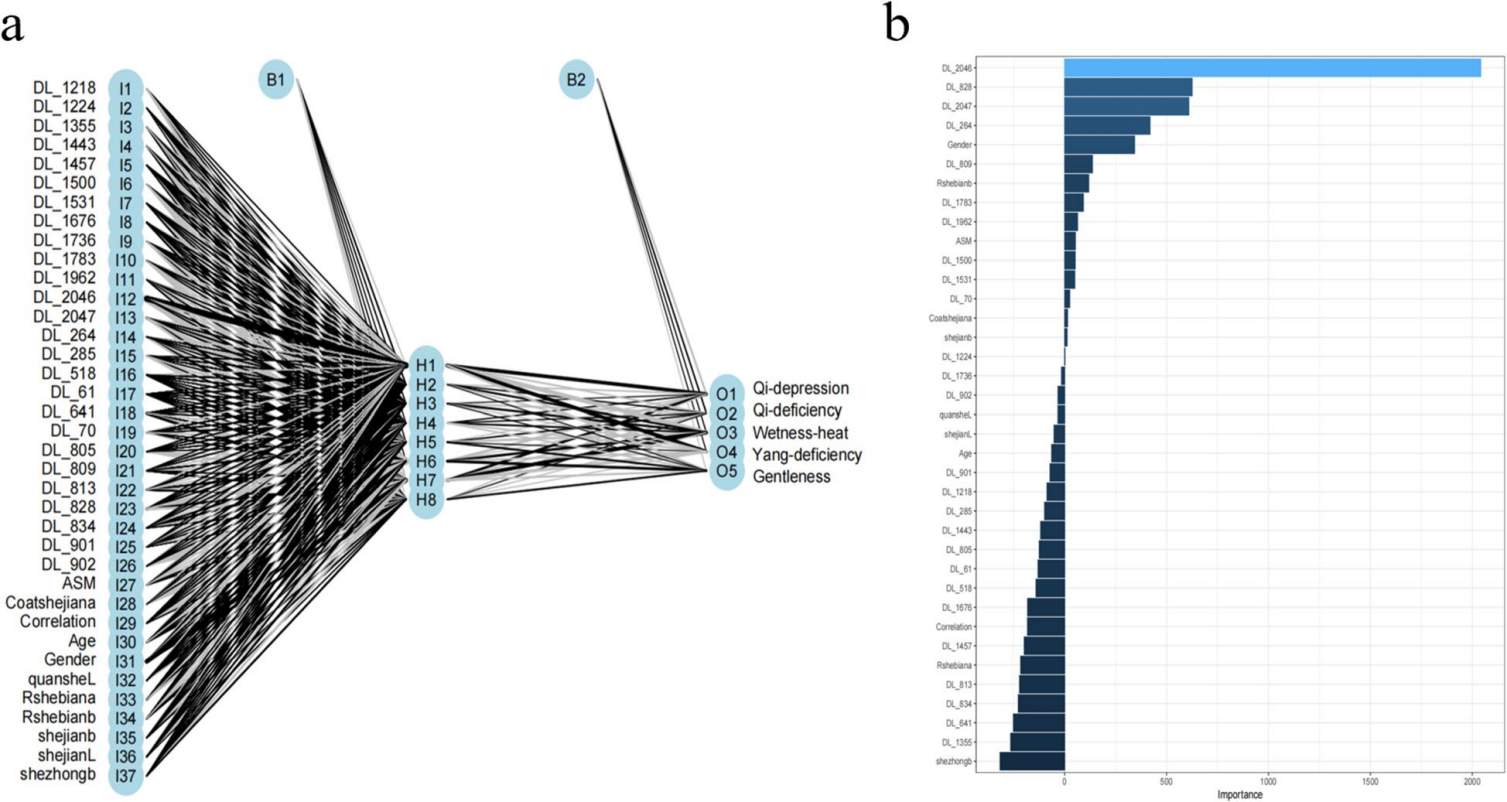
**a** MLP model architecture diagram. **b** Tongue image fusion feature importance ranking

In order to improve the accuracy of TCM constitution recognition, we constructed a multilayer perceptron (MLP) model that integrates traditional features with deep features. The model architecture is shown in Fig. 11a, where the input layer contains multiple nodes (I1 to I37) representing various features extracted from tongue images, including traditional statistical features (e.g., ASM, Correlation, Age, Gender, etc.) and deep learning features (e.g., DL_1218, DL_1224, …, DL_902). These features are fed into two hidden layers (B1 and B2), each with eight nodes (H1 to H8). The output layer contains five nodes (O1 to O5) corresponding to five different TCM body types. After constructing and training the MLP model with this fusion of features, we used the Random Forest algorithm to rank the importance of the features in the model to further optimise the model performance. Figure 11b demonstrates the feature importance ranking results obtained based on the random forest algorithm. The results show that deep learning features (e.g., DL_2046, DL_628, etc.) and traditional features (e.g., gender, age, etc.) have different importance in the model.

**Fig. 12:**
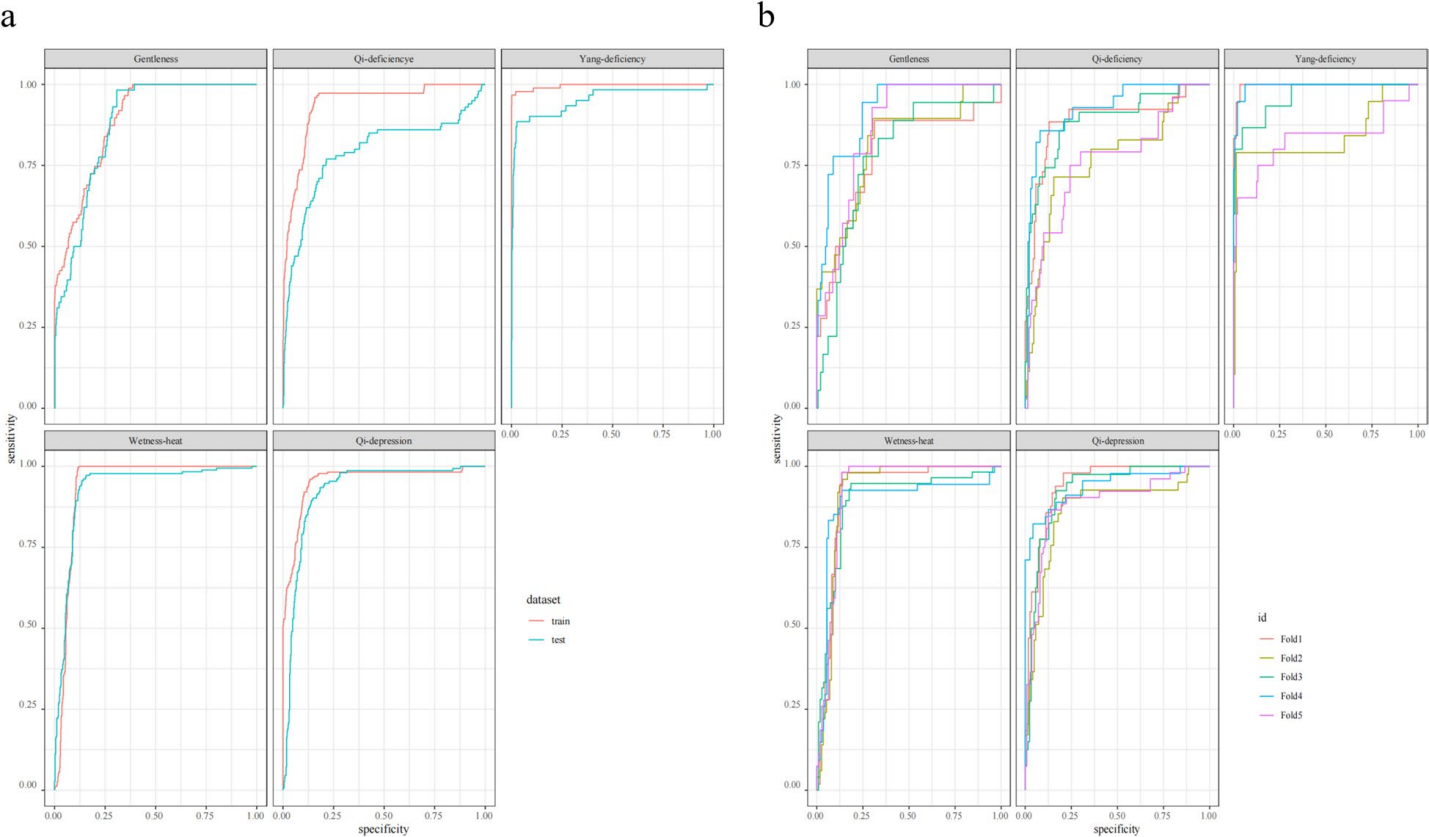
MLP model evaluation index based on tongue image fusion feature. **a** Model ROC curve. **b** Fivefold cross validation

Meanwhile, in order to evaluate the generalisation ability of the MLP model based on tongue fusion features, we divided the collected tongue-somatic data into training set and test set with a ratio of 8:2 and performed a fivefold cross-validation, and the results are shown in Fig. 12. The area under the ROC curve (AUC) for each fold is 0.906 for Fold1, 0.846 for Fold2, 0.890 for Fold3, 0.938 for Fold4, and 0.852 for Fold5. The average AUC is 0.886, and the standard error is 0.0171. Though the performances of Fold2 and Fold5 are slightly lower than the other folds, the overall performance is still good, indicating that the model can still maintain high accuracy and generalisation ability when facing new data, further validating the strong generalisation ability of the model on the test set.

The MLP model based on tongue fusion features shows significant advantages in the field of TCM constitution recognition, which not only improves the overall recognition accuracy, but also improves the recognition accuracy of multiple constitution types while ensuring a lower misdiagnosis rate. Such a model has potential application value in assisting TCM diagnosis and the selection of personalised treatment plans (Table 5).

**Table 5:** Evaluation metrics for train set and internal test set

MLP	Accuracy	Sensitivity	Specificity	AUC	F1	MCC	KAP	ppv	npv
Train set	0.813	0.761	0.949	0.948	0.777	0.753	0.749	0.841	0.955
Test set	0.737	0.680	0.930	0.898	0.680	0.653	0.649	0.703	0.935

### Feature visualization based on grad-cam and analysis of Traditional Chinese Medicine theory

The interpretability of deep learning models has long been a challenge, making the transparency and comprehensibility of model decision-making processes a key issue in research [39]. To address this challenge and enhance the interpretability of our body constitution recognition model, we employed Grad-CAM (Gradient-weighted Class Activation Mapping) technology to generate heat maps [40]. This technique visually illustrates the specific areas of the tongue image that the model emphasizes during body constitution identification, along with their corresponding pathological features as defined in Traditional Chinese Medicine (TCM). This approach not only identifies the characteristic tongue features associated with different body constitution types but also connects these features to specific physiological or pathological mechanisms in TCM, thereby improving the model’s transparency and credibility.

**Fig. 13:**
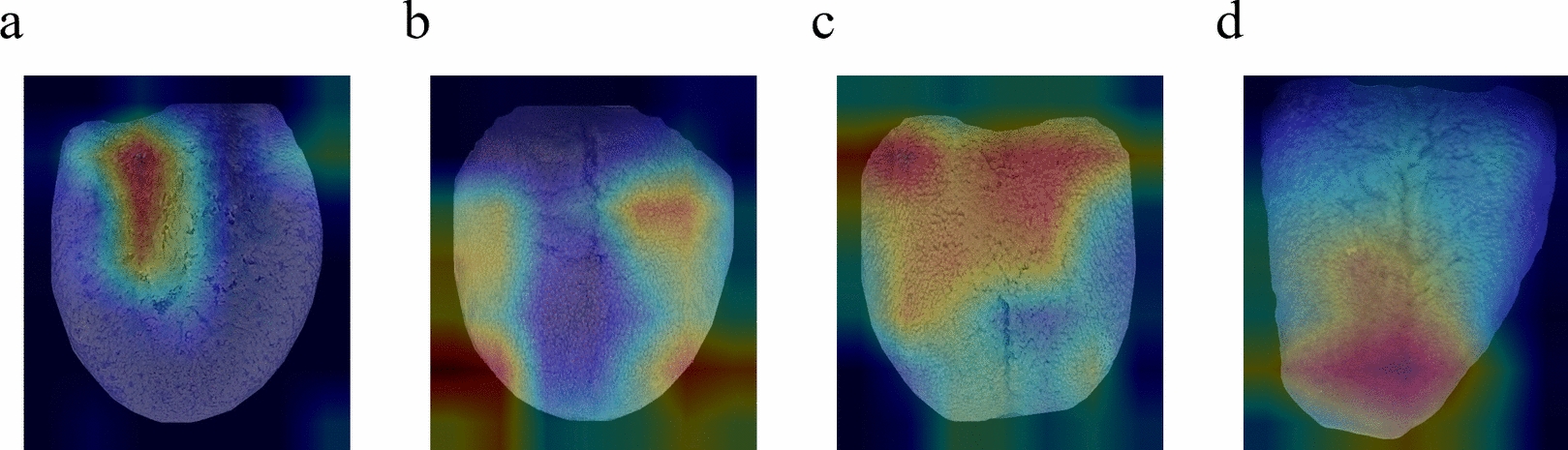
Heat maps of tongue images of different TCM constitution types. **a** Qi-deficiency, **b** Yang-deficiency, **c** Wetness-heat, **d** Qi-depression

The Grad-CAM model highlighted the following features: For Qi-deficiency (Fig. 13a), the model focused on cracks in the central part of the tongue, suggesting spleen and stomach weakness, as well as insufficient Qi and blood production in TCM; for Yang-deficiency (Fig. 13b), it noted tooth indentations along the edges of the tongue, indicating spleen and kidney Yang deficiency with internal dampness; for Wetness-heat (Fig. 13c), the model observed a yellow and greasy coating on the tongue, suggesting the accumulation of damp-heat in the body; and for Qi-depression (Fig. 13d), it highlighted red changes at the tip of the tongue, which in TCM theory are associated with excessive heart fire or liver Qi-depression.

These heat maps not only illustrate the typical tongue features identified by the model for various body constitution types, but they also provide strong support for understanding the underlying TCM theories associated with these features.

## Discussion

In the field of disease diagnosis, treatment and health management, the TCM doctrine of constitution provides an important theoretical basis for individualised health management [41]. This doctrine emphasises the close association between differences in individual constitution and the occurrence, development and regression of diseases, and therefore accurate identification of constitution type is a key link in achieving accurate health management. Tongue diagnosis, as an important part of the four diagnostic methods of traditional Chinese medicine (TCM), has a unique advantage in the identification of constitution by observing tongue characteristics to assess individual health status. However, traditional tongue diagnosis methods mainly rely on physicians’ accumulated experience and subjective judgement, and have obvious limitations in terms of diagnostic consistency and accuracy, which to a certain extent restricts their application in modern health management [42].

With the rapid development of artificial intelligence technology, computer vision provides a new solution for the objectivity and standardisation of tongue diagnosis. By constructing an intelligent mapping model from tongue images to TCM constitution types, deep learning-based image analysis technology can accurately capture the subtle feature differences of the tongue and continuously optimise the model performance through the continuous learning of large-scale data, thus significantly improving the accuracy, reliability and efficiency of constitution identification, while effectively reducing the interference of human factors on the diagnostic results [43]. Ma et al. proposed an improved FlexMatch network method to tackle the issues of limited labeled data and data imbalance [44]. By integrating the attention mechanism with WideResNet and using a focal loss function, they enhanced data utilization efficiency and model performance, achieving an accuracy of 72.9%. Zhao et al. introduced a traditional Chinese medicine constitution classification method based on tongue feature analysis [7]. By collecting tongue images and extracting 45 features, they utilized random forests, LightGBM, and CatBoost to select 39 key features, constructing an ensemble learning model.

Compared to previous studies, our research not only utilised deep learning techniques for precise segmentation of tongue images but also integrated traditional and deep features to select a more streamlined and effective feature set using Lasso regression and random forests. Ultimately, the multilayer perceptron (MLP) model we developed demonstrated significantly superior performance in the classification of constitution compared to models relying solely on a single type of feature. Specifically, during the training phase, our model attained an accuracy (ACC) of 0.893 and an area under the curve (AUC) of 0.948, with sensitivity and specificity values of 0.761 and 0.949, respectively. In the testing dataset, the model achieved an ACC of 0.837, an AUC of 0.898, with a sensitivity of 0.680 and a specificity of 0.930. These results indicate that our model not only surpasses the accuracy and stability demonstrated in Zhao et al.’s research but also exhibits enhanced efficiency and precision when handling complex features. Furthermore, to improve the model's transparency and credibility, we adopted a method inspired by Zhou et al. [45], which involves showcasing key feature maps that illustrate the basis for the model’s constitution classification. This approach not only presents the predicted class scores for any given image but also highlights the identified key object regions, thereby facilitating the visual representation of detection results through heatmaps.

Based on this research result, we further developed a mobile application platform for the Smart Tongue Diagnostic System, which enables users to complete tongue image collection and instantly obtain a body analysis report by themselves at home. The platform will effectively address issues related to lighting interference and user operational errors by integrating automatic exposure adjustment and an intelligent feedback system, thereby ensuring the quality and accuracy of tongue images captured. Furthermore, in light of the high sensitivity of health data, the platform will strictly adhere to relevant laws and regulations to safeguard user data security and privacy. This non-invasive, low-cost, fast and efficient method not only lowers the threshold for accessing professional body mass assessment services, but also enables early intervention and personalised health management. Through the wide application of the mobile platform, the smart tongue diagnosis system significantly improves the efficiency of healthcare services and reduces the consumption of healthcare resources, while promoting the public’s understanding and acceptance of Chinese medicine culture. This study significantly improved the accuracy of TCM constitution identification through feature screening and fusion, injected new vitality into the modern TCM diagnosis and treatment system, and provided an efficient and accurate intelligent identification method for TCM constitution identification.

Despite the results achieved in this study, there are still some limitations that need to be addressed in future work. Firstly, although we employed a rigorous internal validation strategy that included an 80:20 data split and five-fold cross-validation to evaluate model performance, the absence of independent external test set validation limits a comprehensive assessment of the model’s generalisability. To avoid issues related to insufficient training sample size caused by directly splitting external test sets from existing datasets, we plan to continue collecting additional data from other hospitals or community health service centres in future work, thereby constructing an independent external test set. Future research will expand the diversity of the dataset by incorporating tongue image data from a broader range of countries and regions, in order to validate and enhance the model’s applicability across different populations, ultimately improving its potential for global application. Secondly, at this stage, the model is mainly optimised for five common body types, and further validation and improvement is needed for the recognition of other rare body types. Finally, the model performance is highly dependent on high-quality tongue image input, and it is a challenge to ensure consistency and standardisation of image acquisition in practical applications. Future research will aim to expand the dataset, optimise the model to cover more somatic types, and explore simple and reliable image acquisition methods, with a view to improving the overall performance and practicality of the model. Through continuous optimisation and expansion, this study will provide a more solid foundation for intelligent and globalised applications of TCM constitution recognition.

## Conclusions

In this study, the quantitative analysis of traditional features of tongue images and deep features of tongue images was achieved, and a feature fusion-based TCM constitution recognition model was successfully constructed, demonstrating the great potential of deep learning technology in the field of Chinese medicine. With its simplicity, economy and high efficiency, the tongue image-based TCM constitution recognition method opens up a new path to promote the modernisation process of TCM, which is not only conducive to improving the accuracy of individual health management, but also of great significance to optimising the overall allocation of healthcare resources in the society.

## Data Availability

The data and materials in this study are available from the corresponding author upon request.

## Abbreviations

TCM: Traditional Chinese Medicine
CCMQ: the Constitution in Chinese Medicine Questionnaire
CSTCM: the Chinese Society of Traditional Chinese Medicine
GC: gastric cancer
GLCM: Grey Level Co-occurrence Matrix
SDG: Stochastic Gradient Descent
SMOTE: Synthetic Minority Over-sampling Technique
LASSO: Least absolute shrinkage and selection operator
RF: Random Forest
MLP: Multilayer Perceptron
DT: decision tree
KNN: K-nearest neighbour
Multionm: unordered multiclassified logistic regression analysis
Enet: ElasticNet
LightGBM: Light Gradient Boosting Machine
XGBboost: Extreme Gradient Boosting
TP: True Positive
TN: True Negative
FP: False Positive
FN: False Negative
MSE: the mean squared error
ACC: Accuracy
MIoU: Mean Intersection over Union
MCC: Matthews correlation coefficient
KAC: Kappa coefficient
AUC: area under the curve
CI: Confidence Interval
NPV: negative predictive value
OOB: overall error rate
Grad-CAM: Gradient-weighted Class Activation Mapping
